# Diagnostic potential of genomic blood biomarkers of pulmonary fibrosis in a prospective cohort

**DOI:** 10.1371/journal.pone.0314876

**Published:** 2024-12-03

**Authors:** Daniel He, Casey P. Shannon, Jeremy A. Hirota, Kjetil Ask, Christopher J. Ryerson, Scott J. Tebbutt

**Affiliations:** 1 Department of Medicine, Division of Respiratory Medicine, University of British Columbia, Vancouver, BC, Canada; 2 Centre for Heart Lung Innovation, St Paul’s Hospital, Vancouver, BC, Canada; 3 Prevention of Organ Failure (PROOF) Centre of Excellence, Vancouver, BC, Canada; 4 Providence Health Care Research Institute, Providence Research, Vancouver, BC, Canada; 5 Department of Medicine, McMaster University, Hamilton, ON, Canada; 6 Department of Medicine, Firestone Institute for Respiratory Health–Division of Respirology, McMaster University, Hamilton, ON, Canada; 7 Department of Biology, University of Waterloo, Waterloo, Ontario, Canada; Kurume University School of Medicine: Kurume Daigaku Igakubu Daigakuin Igaku Kenkyuka, JAPAN

## Abstract

Fibrotic interstitial lung diseases (ILDs) result from excessive deposition of extracellular matrix (ECM) proteins in the lung, causing irreversible damage to the lung architecture. Clinical management of ILDs differs depending on the diagnosis, but differentiation between subtypes can be difficult and better clinical biomarkers are needed. In this study, we use a 166-gene NanoString assay to investigate whether there are ILD subtype-specific transcripts in whole blood. We identified one transcript, killer cell lectin like receptor 1 (*KLRF1*), as differentially expressed between idiopathic pulmonary fibrosis (IPF) and systemic sclerosis-associated ILD (SSc-ILD), and identified two transcripts (*VCAN*, *LTK*) associated with IPF expression against other ILD subtypes. These findings were validated by examining their expression in ILD lung, with *KLRF1* expression significantly higher in SSc-ILD compared to IPF and hypersensitivity pneumonitis (HP) samples. Taken together, this pilot study provides support for the use of the peripheral transcriptome in identifying diagnostic biomarkers of ILD with biological relevance.

## Introduction

Interstitial lung diseases (ILDs) are a heterogenous group of diseases affecting the lung parenchyma through fibrotic and/or inflammatory processes. In fibrotic ILDs, excess deposition of extracellular matrix (ECM) proteins in the pulmonary interstitium result in distortion of the lung architecture, irreversibly impairing gas exchange and ultimately causing early mortality [1]. Early diagnosis is a major clinical challenge in the management of ILD, and patients with ILD are often at risk of misdiagnosis, inappropriate or delayed treatment, and worse outcomes as a result [2].

Given the scarcity of disease biomarkers in ILD, investigations into molecular biomarkers of ILD (particularly circulating biomarkers) are an ongoing area of interest [3–5]. A 52-gene signature derived from peripheral blood mononuclear cell (PBMC) transcriptomic profiling has been shown to be predictive of transplant-free survival in patients with IPF [6,7], and transcriptomic profiling has also identified genes associated with decline in lung function in the ILD subtypes idiopathic pulmonary fibrosis (IPF) and hypersensitivity pneumonitis (HP) [8–10].

While it is evident that the peripheral transcriptome is associated with prognosis in ILD, to our knowledge, no investigations have been performed regarding its diagnostic potential. The objective of this study was to evaluate the ability of the whole blood transcriptome in discriminating between ILD subtypes. In this pilot study, we profiled whole blood RNA obtained from patients with ILD (n = 59) using a previously published NanoString assay designed to discriminate between the early- and late-phase asthmatic response [11]. Given that fibrosis is a T_H_2-driven process like asthma [12], we reasoned that the transcripts profiled in the assay might also differentiate between other T_H_2-like disease subtypes.

## Materials and methods

### Study population and sample processing

Patients were diagnosed with fibrotic ILD (idiopathic pulmonary fibrosis, IPF, n = 22; fibrotic hypersensitivity pneumonitis, HP, n = 14; systemic sclerosis-associated ILD, SSc-ILD, n = 20; interstitial pneumonia with autoimmune features, IPAF, n = 3) in accordance with diagnostic guidelines and prospectively recruited with written informed consent (ethics protocol H09-00748) between 2012 and 2018 at St. Paul’s Hospital (Vancouver, BC, Canada). At time of sampling, patients were either undergoing anti-fibrotic (pirfenidone, nintedanib), anti-inflammatory (prednisone, mycophenolate, N-acetylcysteine), or no pharmacotherapy. Whole blood samples were collected in PAXgene Blood RNA Tubes (BD Biosciences, Mississauga, ON, Canada) and extracted for RNA using the PAXgene Blood miRNA Kit (PreAnalytiX, Hombrechtikon, Switzerland). RNA quality was confirmed by the RNA 6000 Nano Kit (Agilent, Santa Clara, California, USA) via RNA integrity numbers obtained from Bioanalyzer analysis. Blood transcript expression was profiled using 100 ng of purified RNA with a custom NanoString nCounter Elements assay (NanoString Technologies, Seattle, WA, USA) that measured 166 transcripts [11]. Samples were randomized across six NanoString cartridges (12 samples per cartridge), with remaining assay slots used for sample replicates to assess data reproducibility. Data are available via the Gene Expression Omnibus (GEO) accession number GSE267652.

### RNAscope

Custom RNAscope™ probes for KLRF1 (276–375 of NM_016523.1), LTK (2419–2518 of NM_001135685.1), and VCAN (transcript variant 3; 1356–1455 of uc003kij.3) were developed by Advanced Cell Diagnostics (ACD, Newark, California). Tissue microarray (TMA) sections (4μM-thick) were obtained from a formalin-fixed and paraffin-embedded TMA block containing cores from archived donor lung tissue samples of patients with IPF, HP, and SSc-ILD collected at McMaster University. *In situ* hybridization (ISH) was performed using a Leica BOND RX autostainer with associated Leica reagent kits (Concord, Ontario, Canada) on TMA sections. Slides were scanned at 40X magnification with an Olympus VS120-L100 Virtual Slide System for image capture and a Leica Aperio ScanScope AT2 for quantification. Image analysis was performed using QuPath (v0.4.3), and probe quantification was determined using H-scores as indicated by manufacturer protocols.

### Data analysis

NanoString data underwent quality assessment by examining field of view (FOV) ratio (number of FOV images successfully captured), binding density (image saturation), linearity of positive controls, and limit of detection via negative controls. Data were filtered for lowly abundant features, then normalized using normalization factors derived from the geometric mean of 10 housekeeping transcripts. Differential expression analysis of transcripts was performed using the linear models for microarray (limma) R package (v3.54.2), while biomarker panels were developed using sparse partial least squares discriminant analysis (sPLSDA), elastic net, and random forest algorithms from the ‘mixOmics’ (v6.22.0) and ‘tidymodels’ (v1.0.0) R packages. Image analysis was performed using QuPath (v0.4.3) by automatically identifying individual cells and setting probe-specific thresholds based on signal strength. Cells without a visible nucleus were excluded from analysis. H-scores were determined by grouping cells into 5 bins based on the numbers of dots per cell (0, 1–3, 4–9, 10–15, >15) and multiplying the total percentage of cells in each bin by 0 to 4. To test for statistical significance of H-scores, a linear mixed-effects model (‘lme4’ v1.1–32) was fitted with the formula H-Score ~ Diagnosis + (1|Patient) and pairwise comparisons between diagnoses to determine statistical significance with Tukey’s post-hoc adjustment were performed using the ‘emmeans’ (v1.8.5) R package. Fisher’s exact test was used to test for differences in demographic count variables between diagnoses, while the F-test was used to test for differences in demographic continuous variables between diagnoses. All analysis was performed in the R (version 4.2.3) statistical computing environment.

## Results

We profiled the expression of 166 RNA transcripts in a cohort of patients with IPF, systemic sclerosis-associated ILD (SSc-ILD), HP, or interstitial pneumonia with autoimmune features (IPAF) (Table 1). No significant differences were identified between subtypes for race, percent predicted forced vital capacity (FVC%) or diffusing capacity of the lung for carbon monoxide (D_L_CO%), smoking status, or treatment status, but patients with SSc-ILD and IPAF were younger at time of sampling and patients with IPF were predominantly male which is in line with previously reported cohorts [13].

**Table 1:** Summary table of clinical demographics of patients probed for whole blood RNA expression.

	IPF (N = 22)	SSc-ILD (N = 20)	HP (N = 14)	IPAF (N = 3)	P-value
Male sex, n (%)	17 (77)	2 (10)	5 (36)	1 (33)	***P* < 0.0001**
Age, mean ± SD	69.91 ± 7.31	60.85 ± 9.12	66.21 ± 7.26	56.00 ± 7.81	***P* = 0.0014**
Race, n (%)					*P* = 0.6358
Asian	2 (9)	4 (20)	1 (7)	1 (33)	
American Indian	1 (5)	0 (0)	0 (0)	0 (0)	
Pacific Islander	1 (5)	0 (0)	1 (7)	0 (0)	
White	18 (82)	16 (80)	12 (86)	2 (67)	
FVC% predicted, mean ± SD	74.18 ± 15.29	83.10 ± 18.07	70.21 ± 16.30	72.67 ± 20.50	*P* = 0.1435
D_L_CO% predicted, mean ± SD	42.41 ± 11.79	54.70 ± 19.46	45.00 ± 13.21	52.00 ± 5.20	*P* = 0.0624
Ever smoked, n (%)	16 (80)	12 (60)	7 (50)	2 (67)	*P* = 0.2780
Treatment, n (%)					*P* = 0.6981
Anti-Fibrotic	9 (41)	0 (0)	0 (0)	0 (0)	
Anti-Inflammatory	0 (0)	11 (55)	9 (64)	2 (67)	
Anti-Fibrotic and Anti-Inflammatory	1 (5)	0 (0)	0 (0)	0 (0)	
None	12 (55)	9 (45)	5 (36)	1 (33)	

**Fig 1:**
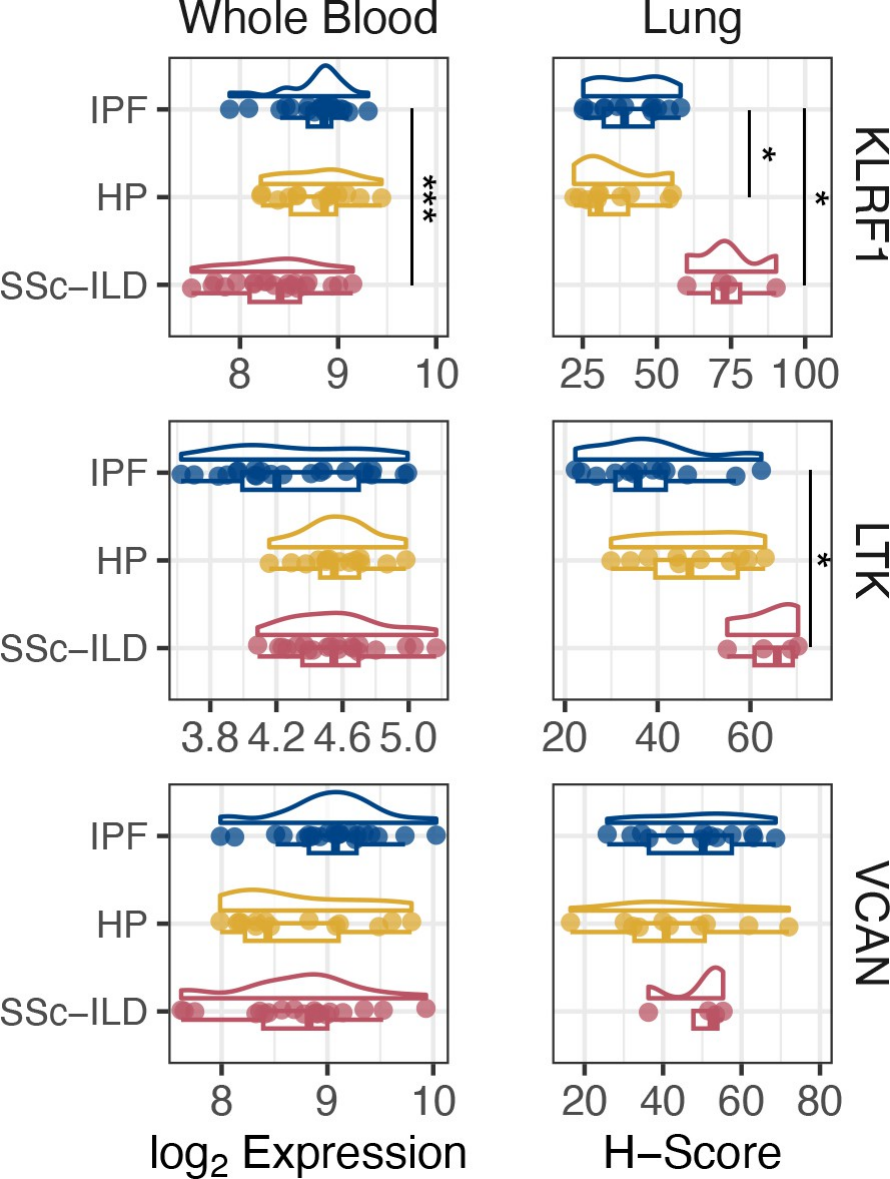
Expression of *KLRF1*, *LTK*, *VCAN* in whole blood (log_2_ RNA expression) or lung tissue microarray sections (H-Score) containing samples from patients with idiopathic pulmonary fibrosis (IPF), hypersensitivity pneumonitis (HP), and systemic sclerosis-associated interstitial lung disease (SSc-ILD). Statistical significance of blood RNA expression was determined using differential expression analysis via ‘limma’ and corrected using false discovery rate (FDR), with statistical significance indicated by FDR<0.05 (***). To determine statistical significance of H-scores, a linear mixed-effects model was fitted and pairwise comparisons between diagnoses were performed and corrected for using Tukey’s post-hoc adjustment (* denotes *p*<0.05).

To identify variables that might affect RNA expression, we performed a principal component (PC) analysis and correlated the PCs with demographic variables (S1 Fig). After adjusting for race and treatment status, we identified 1 differentially expressed transcript at an FDR of 0.05 when comparing IPF to SSc-ILD (*KLRF1*, FDR = 0.03) (S1 Table and Fig 1). While no transcripts were significantly associated with FVC%, we identified 13 transcripts that were significantly associated with D_L_CO% (S1 Table). Next, we examined the utility of RNA profiling in developing biomarker panels to differentiate between ILD subtypes through the use of three learning algorithms (sparse partial least squares discriminant analysis (sPLSDA), elastic net, and random forest). The best-performing models using 5-fold cross-validation repeated ten times were IPF vs SSc-ILD (sPLSDA) and IPF vs SSc-ILD (elastic net), which yielded respective accuracies of 0.74 ± 0.01 and 0.80 ± 0.02 (S2 Table).

**Fig 2:**
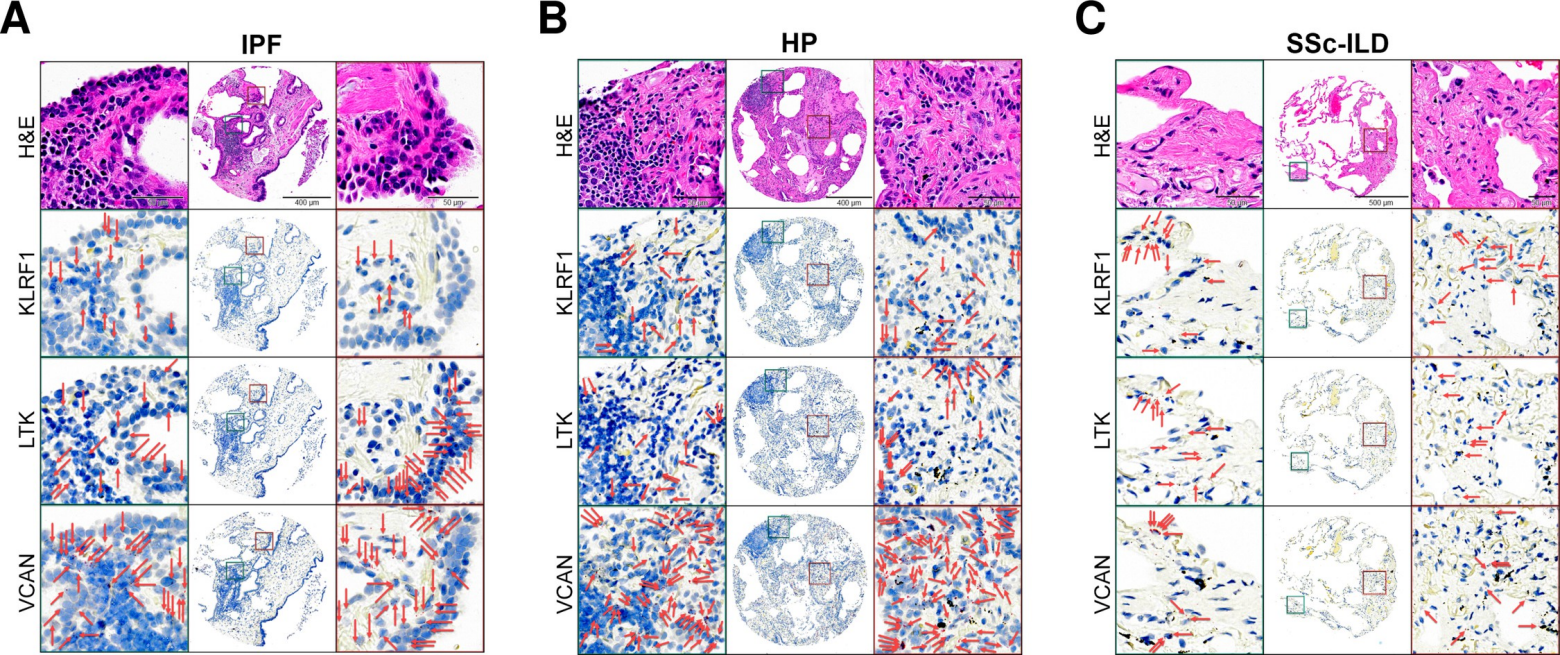
Histological staining of tissue microarray cores taken from donor lungs of patients with idiopathic pulmonary fibrosis (IPF), hypersensitivity pneumonitis (HP), and systemic sclerosis-associated interstitial lung disease (SSc-ILD). Arrows indicate areas of positive staining.

To investigate the lung expression of the three most highly weighted genes (*KLRF1*, *LTK*, *VCAN*) identified by our biomarker panels when comparing IPF vs non-IPF ILD (S3 Table), we used ISH on TMA sections consisting of twelve cores from five patients with IPF, ten cores from three patients with HP, and four cores from two patients with SSc-ILD (Fig 2), all of which showed no significant between-group differences in demographic or clinical variables (Table 2). Transcript abundance was determined semi-quantitatively by calculating H-scores for each marker per core, which is a weighted total of the percentage of cells expressing the probe at varying levels. Using a linear mixed-effects analysis, the H-score of *KLRF1* was higher in SSc-ILD compared to IPF (*p* = 0.02) and HP (*p* = 0.01), while *LTK* was lower in IPF compared to SSc-ILD (*p* = 0.03) (Fig 1).

**Table 2:** Summary table of clinical and demographic characteristics of patients with ILD profiled for lung ISH. Data for race, DLCO%, and treatment status was not available.

	IPF (*n* = 5)	HP (*n* = 3)	SSc-ILD (*n* = 2)	P-value
Female sex, n (%)	4 (80)	3 (100)	1 (50)	*P* = 0.2222
Age, mean ± SD	59.40 ± 9.32	59.67 ± 10.79	62.00 ± 9.90	*P* = 0.9182
FVC% predicted, mean ± SD	52.00 ± 12.81	72.67 ± 5.13	1; 80.00 ± NA	*P* = 0.3523
Ever smoked, n (%)	2 (40)	2 (67)	1 (50)	*P* = 0.7143

## Discussion

The 2018 ATS/ERS/JRS/ALAT diagnostic guidelines for IPF made a recommendation to investigate novel molecular biomarkers that could be integrated into clinical diagnosis [14]; in the 2022 update, a recommendation was not made for or against a genomic classifier identifying a usual interstitial pneumonia pattern diagnostic of IPF in transbronchial biopsies, thus highlighting the continued need for research into molecular biomarkers [15]. Although previous studies have investigated the peripheral transcriptome in relation to prognosis [6,8,10], no study has looked at its diagnostic potential. In this pilot study, we demonstrate that the peripheral blood RNA can differentiate between ILD subtypes, in particular IPF and SSc-ILD, through differential expression and learning algorithms utilizing gene expression data obtained from a custom NanoString assay. The blood gene expression data show association with DLCO% predicted but not FVC% predicted, which was previously noted when examining differential expression of the peripheral transcriptome in mild and severe IPF [8]. Furthermore, we identify *KLRF1* expression as a significant in the differentiation of IPF and SSc-ILD in both whole blood and lung, and down-regulation of *LTK* as being associated with IPF.

Killer cell lectin like receptor F1 (KLRF1, also known as NKp80) is expressed in T cells, monocytes, and CD56^bright^ natural killer (NK cells), and is a marker of NK cell maturity [16–18], inducing cytotoxic activity and calcium mobilization [19]. The dysfunction of NK cells in systemic sclerosis and autoimmunity has been well-documented [20–22], and differences in their abundance have been observed between ILD subtypes [23,24]. We found that *KLRF1* expression was associated with lower D_LCO_%, and its expression was lower in the peripheral blood of patients with SSc-ILD compared to IPF and HP yet the opposite effect was observed when probing lung expression. Previous reports have identified a reduction in abundance and function of NK cells in IPF lungs concurrent with an increase in abundance in blood compared to healthy controls [25]. Single-cell RNA-sequencing data available via the IPF Cell Atlas [26] suggests higher *KLRF1* expression in SSc-ILD T/NK subsets compared to other ILDs. Given the disparity between single-cell and bulk RNA data, further investigations are required to delineate the mechanisms surrounding NK cell recruitment to the ILD lung and its role in ILD pathogenesis.

*LTK* and *VCAN* were differentially expressed at a nominal *p*-value of 0.05 between IPF and non-IPF ILDs (but not at FDR<0.05), and were two highly weighted coefficients in our elastic net and sPLSDA biomarker panels. *LTK* is highly expressed in plasmacytoid dendritic cells (pDCs) (Human Protein Atlas, IPF Cell Atlas), and decreased circulating pDCs have been reported in IPF patients [27], which is consistent with the decreased *LTK* expression observed in this study in blood and lung. *VCAN* encodes for versican, an ECM proteoglycan that is localized to fibroblastic foci in the lung, myofibroblasts, and monocytes [26,28]. We were unable to identify a statistically significant increase in *VCAN* expression in IPF compared to HP or SSc-ILD lung and blood samples; however, given its detectable expression in both lung and peripheral blood, and prior studies showing circulating levels of versican degradation products are associated with increased mortality in idiopathic ILDs [29], further studies into its gene expression as a prognostic biomarker may be warranted.

In this pilot study, we report the first between-subtype comparison of the peripheral ILD transcriptome using a discrete set of genes, which validates performing more extensive genome-wide analysis of peripheral ILD subtype-specific profiles and correlations of identified biomarkers in lung tissue. In particular, molecules detected in the blood that are associated with immunity and fibrosis are detectable in the lung, thereby suggesting a test based on peripheral transcriptomics is feasible for diagnostic biomarkers with biological relevance, similar to prognostic markers in Herazo-Maya *et al*. [7]. Given the heterogeneity within ILD subtypes, future analysis of the peripheral transcriptome should also consider radiological pattern, progressive phenotype, and other related clinical factors [30].

## Supporting information

S1 Table List of transcripts analyzed for differential expression between ILD subtypes and with lung function. Sheets with “_cov” denote comparisons made after adjusting for race and treatment status.
(XLSX)

S2 Table Classification model performance on cross-validation for selected learning algorithms. Data are reported as accuracy ± standard error.
(CSV)

S3 Table Coefficient weights of biomarker panels developed for each between subtype comparison. (XLSX)

S1 Fig Correlation of principal component analysis (PCA) components (PCs) with demographic and clinical metadata. Values shown are Pearson correlation coefficients.
(TIF)
