# Supplementary figures and images for: Diagnostic potential of genomic blood biomarkers of pulmonary fibrosis in a prospective cohort
Source: PLoS One. 2024 Dec 3;19(12):e0314876. doi: 10.1371/journal.pone.0314876 (PMC11614250; doi:10.1371/journal.pone.0314876)

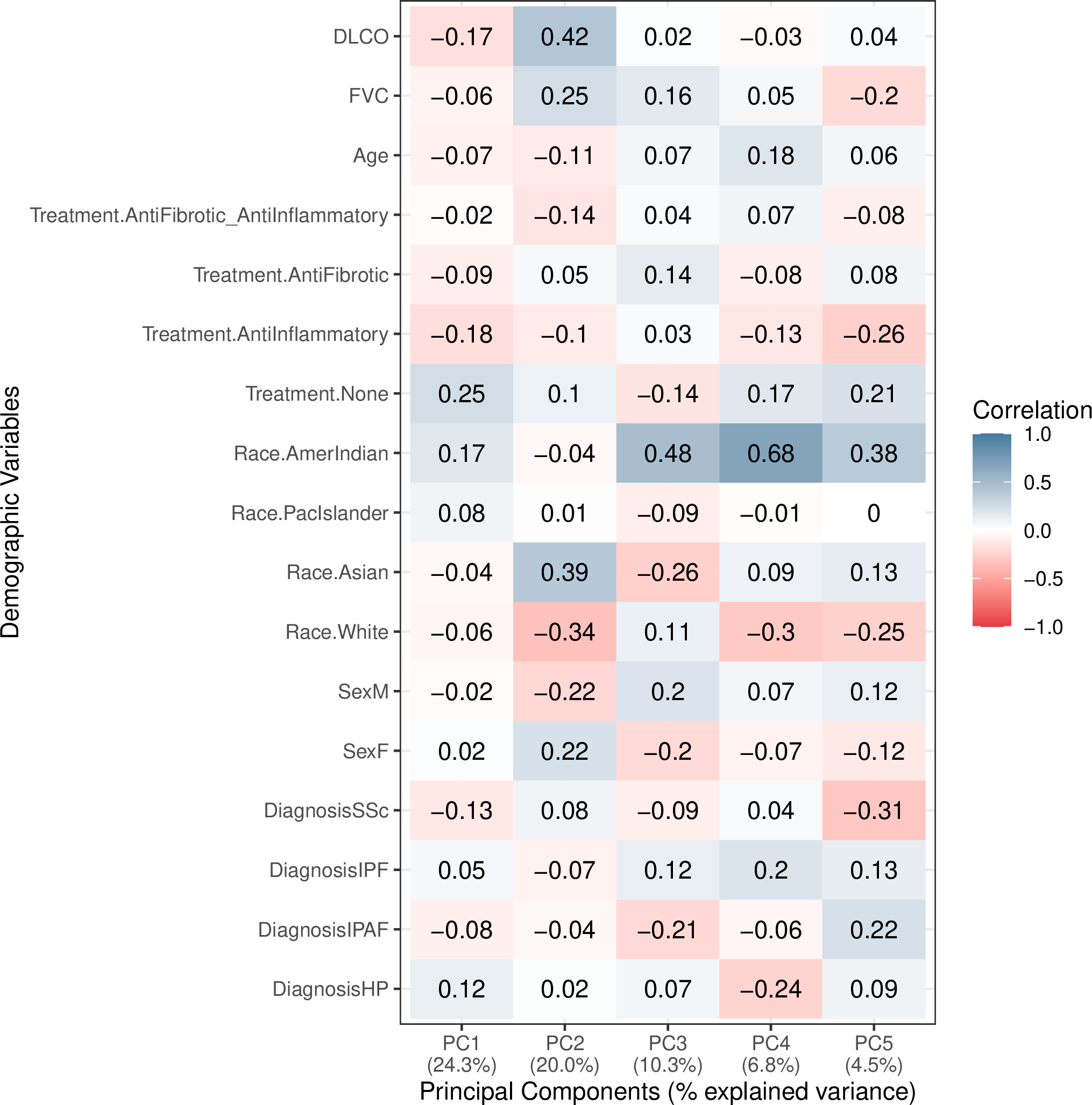

Supplement: S1 Fig — Values shown are Pearson correlation coefficients. (TIF) [file pone.0314876.s004.tif]
